# Cocaine Upregulates Microglial Lipid Droplet Formation Through Increasing Lipid Synthesis Activity In Vitro and In Vivo

**DOI:** 10.3390/biom16040526

**Published:** 2026-04-01

**Authors:** Yan Cheng, Brooke Russell, Liam Liyang Guo, Aryan Patel, Yan Y. Sanders, Ming-Lei Guo

**Affiliations:** 1 Department of Biological and Translational, Macon and Joan Brock Eastern Virginia Medical School/Virginia Health Sciences, Old Dominion University, Norfolk, VA 23507, USA; chengy@odu.edu (Y.C.); b1russel@odu.edu (B.R.); aryan.patel4@outlook.com (A.P.); sandery@odu.edu (Y.Y.S.); 2 Keck School of Medicine, University of Southern California, Los Angeles, CA 90033, USA; liyanggu@usc.edu; 3 Center for Integrative Neuroscience and Inflammatory Diseases, Eastern Virginia Medical School/Virginia Health Sciences, Old Dominion University, Norfolk, VA 23507, USA

**Keywords:** lipid droplets, microglia, neuroinflammation, cocaine, senescence

## Abstract

A novel subtype of microglia, lipid droplet accumulation microglia (LDAMs), has been identified in the aged brain, which is characterized by sustained inflammation and senescent-like phenotypes. LDAMs are deeply involved in promoting brain aging as well as the pathogenesis of multiple neurodegenerative diseases. Cocaine can alter brain lipidomic profiles, induce microglial activation, and accelerate brain aging. This suggests that cocaine might affect microglial lipid metabolism, which ultimately aggravates aging-related neurological disorders in people with addictions. In this study, we explored the effects of cocaine on microglial lipid metabolism. Our results showed that chronic cocaine administration altered brain lipid profiles and increased LDAM formation in vivo. The increase in LDAMs was accompanied by the upregulation of the senescent marker p53 in the brain. Cocaine also increased lipid droplet (LD) formation in BV2 microglia and primary microglia in vitro. A mechanism study revealed that cocaine increased the levels of SREBP1/2, HMGCR, DGAT1, and FASN, which are critical for lipid synthesis. Overall, our findings demonstrate that cocaine increases LDAM formation in vitro and in vivo. These results indicate that targeting microglia lipid metabolism might be a promising therapeutic approach to mitigate aging-related neurological syndromes in people with cocaine addictions.

## 1. Introduction

Microglia, the brain-resident macrophages, constitute a critical component in the first-line-of-defense system that performs constitutive immune surveillance in the central nervous system and have multiple functions as housekeepers, guards, and defenders to maintain brain homeostasis and ensure normal brain functions [1,2,3,4,5]. Microglia are known to be heterogeneously distributed throughout the brain and can be grouped into different subtypes based on their transcriptional profiles from single-cell RNA sequencing. For example, disease-associated microglia have been identified in the brains of mouse models with Alzheimer’s disease and Parkinson’s disease [6,7]. Other subtypes, such as proliferation-associated microglia, neurodegeneration-associated microglia, LDAMs, etc., have also been identified in various brain disease models [8,9,10]. Among these subtypes, LDAMs have been demonstrated to play essential roles in promoting brain aging and the pathogenesis of multiple neurodegenerative diseases [11,12,13]. At the cellular level, LDAMs are characterized by sustained activation, impaired phagocytosis and surveillance, and autophagy dysregulation [11,12,13].

Accumulating evidence shows that microglia can be activated by lipids and free fatty acids (FAs) (immunometabolism) [14,15,16,17]. Lipid droplets (LDs) have been suggested as structural markers for microglial activation. LDs are intracellular organelles with a core of neutral lipids, including triacylglycerols (TAGs) and cholesteryl esters, surrounded by a monolayer of charged phospholipids and proteins [18,19]. Perilipins (PLINs), located outside of the monolayer, serve as markers for LD formation [20]. LD formation is regulated by multiple processes including lipid synthesis, influx and efflux, and degradation [21]. Sterol regulatory element-binding proteins (SREBPs) are the master regulators of lipid, cholesterol, and FA synthesis and are highly conserved between species [22,23]. SREBPs and SREBP-mediated lipid synthesis activity are critical for LD formation. The SREBP family consists of three members: SREBP1a and SREBP1c are responsible for regulating FA metabolism and SREBP2 primarily regulates cholesterol synthesis through increasing the expression of HMG-CoA reductase (HMGCR), which is the rate-limiting enzyme in cholesterol synthesis [24]. At the subcellular level, SREBPs are mainly located in the endoplasmic reticulum (ER) membrane. The activity of SREBPs can be regulated at multiple levels including the transcriptional, post-transcriptional, and post-translational levels. When there are sufficient nutrients (intracellular cholesterol, lipids, and FAs), SREBPs are bound to SREBP cleavage-activating protein and insulin-induced gene 1 and kept in an inactive state. When these nutrients become limited, SREBPs are transferred to the Golgi apparatus and sequentially cleaved by two proteases (site 1 and site 2 proteases), releasing the amino-terminal SREBPs into the nucleus, now called nuclear SREBPs (nSREBPs). The nSREBPs then bind to the promoter regions of downstream genes and activate their transcription; these genes include diacylglycerol O-acyltransferase 1/2 (DGAT1/2) and fatty acid synthase (FASN), which are all critical enzymes for lipid droplet synthesis [22].

Abused drugs can dysregulate energy metabolism, contributing to the pathogenesis of substance use disorders [25,26]. Cocaine modulates polyunsaturated FA status [25,26] and increases brain phospholipid precursors in people with addictions [27]. Cocaine has been shown to induce lipidomic changes in the rat hippocampus (HP) [28]. Chronic cocaine administration also reduces phospholipase A activity in the rat brain striatum [29] and significantly modifies brain lipid profiles in the ventral striatum [30]. Methamphetamine (Meth) and morphine are capable of remodeling the brain lipidome in mice [31,32]. Meth also accelerates cellular senescence through increasing ceramide biosynthesis [33]. Atorvastatin, a drug that inhibits cholesterol synthesis, has beneficial effects on morphine-induced tolerance and dependence in mice [34]. Alcohol can dysregulate lipid peroxidation and metabolism, leading to neurological damage [35,36]. Overall, alterations to lipid metabolism in the brain seem to be a common downstream event induced by various types of abused drugs. However, the cell-type-specific effects of abused drugs on lipid metabolism and the underlying mechanisms remain mostly unknown.

In this study, we explored the effects of cocaine on LDAM accumulation and the underlying mechanisms. Our results showed that cocaine altered the brain lipidomic profile and increased LDAM formation in vitro and in vivo. The increase in LDMAs occurred simultaneously with upregulation of senescent markers in the brain. Mechanistically, cocaine increased lipid synthesis by enhancing the activity of SREBP2-mediated pathways. Overall, our findings suggest that a novel form of immunometabolism is involved in cocaine-mediated microglial activation and senescence. Since microglia senescence is closely associated with brain aging, targeting lipid synthesis and LDAM removal could be a novel therapeutic approach for mitigating aging-related neurological syndromes in people with cocaine addictions.

## 2. Materials and Methods

Animals and reagents: C57/BL6 wild type (WT) mice (male, 3–5 months) were maintained in the animal facility of Virginia Health Sciences (VHS) at ODU. They were housed in a colony room and provided food and water ad libitum. The colony room was maintained with a 12:12 light/dark cycle and an ambient temperature of 24.0 °C ± 1.5 °C. All procedures were conducted in accordance with the National Institutes of Health’s Guide for the Care and Use of Experimental Animals and were approved by the VHS Institutional Animal Care and Use Committee (protocol number: 24-006). BODIPY™ 493/503 was purchased from Thermo Scientific (D3922, Waltham, MA, USA) and dissolved in DMSO to create a stock solution with a concentration of 2 mM, which was diluted to 2 µM to prepare the working solution. Cocaine was purchased from Sigma-Aldrich (C5776, Saint Loius, MO, USA). SREBP2-siRNA and the control siRNA were purchased from Horizon Discovery (Dharmacon^TM^, On-targetplu^TM^ 2.0, Cambridge, MA, USA). Human microglial cells 3 (HMC3) were purchased from ATCC (CRL-3304, Manassas, VA, USA). For cocaine administration, WT mice (3–5 months, male) were injected at a dose of 15 mg/kg for three weeks (i.p. daily).

Lipidomic analysis: The lipidomic analysis was performed by Creative Proteomics. The hippocampus (HP) was dissected from the brain and around 100 mg of tissue (±cocaine, *n* = 4) was mixed with 1.5 mL of 2:1 chloroform:MeOH (*v*/*v*) and ground for 180 s at 65 Hz; then, 0.5 mL of ultrapure water was added and the mixture was sonicated for 30 min at 4 °C. The tubes were then centrifuged for 10 min at 3000 rpm (4 °C), and the lower phase was transferred to a new tube and dried under a nitrogen atmosphere. The dried extract was resuspended with 200 μL of 1:1 isopropyl alcohol:MeOH (*v*/*v*) and 5 μL of LPC (12:0) was added as an internal standard. The tubes were centrifuged for 10 min at 12,000 rpm (4 °C) and the supernatant was aspirated and used for LC-MS analysis. The separation was performed using UPLC (Waters). The LC system comprised an ACQUITY UPLC BEH C18 (100 mm × 2.1 mm, 1.7 μm) column. The mobile phase was composed of solvent A (60% CAN + 40% H_2_O + 10 mM HCOONH_4_) and solvent B (10% CAN + 90% isopropyl alcohol + 10 mM HCOONH_4_), which was used to perform gradient elution (0–1.0 min: 30% B; 1.0–10.5 min: 30–100% B; 10.5–12.5 min: 100% B;12.5–12.51 min: 100–30% B; 12.51–16 min: 30% B). The flow rate of the mobile phase was 0.3 mL/min. The column temperature was maintained at 40 °C, and the sample temperature was set at 4 °C. The raw data were acquired and aligned using the Lipid Search software (Thermo, Version 5.1) based on the *m*/*z* value and the retention time of the ion signals. Irons from both ESI− or ESI+ were merged and imported into the SIMCA-P program (version 14.1) for multivariate analysis. Principal Component Analysis (PCA) was first used as an unsupervised method for data visualization and outlier identification. Supervised regression modeling was then performed on the data set through Partial Least Squares Discriminant Analysis (PLS-DA) or Orthogonal Partial Least Squares Discriminant Analysis (OPLS-DA) to identify the compounds showing significant differences (filter criteria: VIP value > 1.5, *p* value < 0.05, and FC > 2). Univariate analyses, including fold change analysis and *t*-tests, were performed on the volcano plot. Hierarchical cluster analysis (HCA) and pathway analysis based on KEGG databases were also performed to identify dysregulated lipids and pathways relevant to lipid metabolism when comparing the saline- and cocaine-treated groups.

Adult microglia isolation: After cocaine/saline treatment, the mice were anesthetized with 4% isoflurane and transcardially perfused with 1× PBS, followed by brain removal. The brains were pooled using MACS dissociation kits (Miltenyi Biotech Company, Bergisch Gladbach, Germany). Briefly, the brains were homogenized in 2 mL of the enzyme mixture using a gentleMACS™ Octo Dissociator at 37 °C for 30 min (Miltenyi Biotec, Auburn, CA, USA). The homogenates were then transferred to MACS^®^ Smart Strainers (Miltenyi Biotec, Auburn, CA, USA) and centrifuged at 300× *g* for 10 min at 4 °C to remove debris and red blood cells. The pellets were dissolved in 500 µL of the labeling solution. The acquired cells were incubated with 15 µL of CD11b beads for 15 min at 4 °C and were sent through a column in a magnetic field. The purified microglia were suspended in 1 mL of 1× PBS with 0.5% BSA and quantified using a Countess 3 cell counter (Thermo Fisher, Waltham, MA, USA).

Primary microglial and BV2 cell culture: Primary microglia (PMs) were obtained from C57BL/6 newborn pups (<24 h). The brains were removed and cut into small pieces in Hank’s buffered salt solution (Invitrogen, Carlsbad, CA, USA, 14025076) and incubated in 0.25% trypsin (Invitrogen, 25300-054) for 15 min. After filtration with a 70 µM strainer, the mixed glial cultures were resuspended in DMEM (Invitrogen, 11995-065) with 10% heat-inactivated FBS (Invitrogen, 16000-044), 100 U/mL penicillin, and 0.1 mg/mL streptomycin. We seeded the PMs into 75 cm^2^ cell culture flasks at a density of 20 × 10^6^ cells/flask. The cell medium was replaced every 3 days, and after the first medium change, 0.25 ng/mL macrophage colony-stimulating factor (Invitrogen, PHC9504) was added. When confluent (around one week later), the mixed glial cultures were shaken by hand to promote microglia detachment from the bottom of the flask. We then collected the floating PMs from each flask and centrifuged them at 500 *g* for 5 min. The PMs were plated into 6-well plates for all experiments. The purity of the microglial cultures was evaluated by Iba1 immunostaining (Wako Pure Chemical Industries, Osaka, Japan, 019-19741).

The BV2 cells were grown and routinely maintained in DMEM with 10% FBS at 37 °C and 5% CO_2_ and used up to passage 30. BV2 cells or PMs were seeded into 6-well plates at 80% confluence and cultured overnight. The next day, the medium was replaced with fresh DMEM (FBS-free) for 2 h to starve them and then they were exposed to cocaine at varying doses (1–100 µM) for 24 h or a dose of 10 µM for different periods of time (2–24 h). At the indicated time points, BV2 cells or PMs were collected for protein extractions or fixed with 4% PFA for BODIPY immunostaining.

BODIPY and β-gal staining: BODIPY 493/503 is a highly lipophilic, bright green, fluorescent dye used to specifically stain neutral lipids within intracellular LDs. Microglial cells were seeded into chamber slides overnight. After the treatment, the cells were washed with PBS and incubated with a BODIPY solution (2 µM) for 15 min at 37 °C. After washing three times with PBS, the cells were fixed with 4% PFA for 30 min at room temperature. The cells were washed with 1× PBS again and covered with prolong gold antifade reagent with 4,6-diamidino-2-phenylindole and a coverslip. The lipid droplets were immediately imaged using fluorescent microscopy. The β-gal staining procedure followed the protocol recommended by the manufacturer (Invitrogen, Cat # K146501).

Flow cytometry analysis: Adult microglia isolated from saline/cocaine-treated brains or BV2 cells were washed with PBS and incubated with a BODIPY solution (2 µM) in the dark for 15 min at 37 °C. The cells were rinsed with PBS and centrifuged at 500× *g* for 5 min and then resuspended in 5 mL of PBS. The cells were centrifuged at 250× *g* for 5 min at 4 °C twice, and then resuspended in 300 µL of 1× flow cytometry buffer. Flow cytometry analyses were performed using a BD Accuri™ C6 Plus Flow Cytometer System and at least 10, 000 cells in each condition were included for analysis by FlowJo software v10.

Western blots: Brain tissues or microglia cells were dissolved in RIPA buffer with proteinase and phosphatase inhibitors (Thermo Scientific) and sonicated for 10 s on ice at 70% amplitude (Thermo Scientific). The brain homogenates were then incubated at 4 °C for 30 min, followed by centrifugation at 12,000 rpm for 10 min. The supernatants were removed and their protein concentrations were determined using the BCA method. Equal amounts of proteins (25 µg) were electrophoresed in a sodium dodecyl sulfate–polyacrylamide gel (160 V, 60 min) under reducing conditions, followed by transfer to PVDF membranes (180 mA, 90 min). The blots were blocked with 3% nonfat dry milk in Tris-buffered saline (TBST). The membranes were then incubated with the indicated antibodies overnight at 4 °C. The next day, the membranes were washed and incubated with the appropriate IRDye fluorescent mouse or rabbit secondary antibody for one hour at room temperature. After three washes with TBST, the membranes were put into the Odyssey^®^ Imaging System for image development, and the intensity of the fluorescent bands was quantified using Image Studio™ Software (version 6.1). After imaging, the membranes were re-probed with β-actin for normalization. The following antibodies were used: PLIN2 (1:2000, Proteintech, 15294-1-AP); SREBP1 (1:2000, Novus Biological, NB100-2215), SREBP2 (1:2000, Novus Biological, NB100-74543); HMGCR (1:2000, Novus Biological, NBP2-66888); DGAT1 (1:2000, Proteintech, 11561-1); FASN (1:2000, Proteintech, 10624-2); p53 (1:2000, Proteintech, 60283-2-Ig), p21 (1:2000, Proteintech, 28248-1-AP), and β-actin (1:2000, Santa Cruz, sc-8432 or 1:2000, Sigma, A2066). The following second antibodies were purchased from the Li-COR company: IRDye^®^ 680RD donkey anti-mouse (1:5000) and rabbit IgG, and IRDye^®^ 800CW donkey anti-mouse and rabbit IgG (1:5000).

Brain cryosections and double immunofluorescence staining: Mice were anesthetized with 4% isoflurane and transcardically perfused with 1× PBS, followed by fixation with 4% PFA. The brains were removed and put into a 30% sucrose PBS solution overnight. One day later, the brains were cut into 30 µM sections using a cryostat machine (Leica Biosystems, Vista, CA, USA). The brain sections were incubated with primary anti-AIF1/Iba1 (1:1000, Wako Pure Chemical Industries, Osaka, Japan, 019-19741), anti-GFAP (1:1000, ab7260, abcam), or anti-NeuN antibody (1:500, Proteintech, 26975-1-AP) overnight at 4 °C. Secondary AlexaFluor 488 goat anti-rabbit IgG (A-11008) or AlexaFluor 594 goat anti-mouse (A-11032) antibody (Thermo Fisher Scientific, Waltham, MA, USA) was added for 2 h to detect Iba1, GFAP, or NeuN. Then, the sections were incubated with a BODIPY solution (2 µM) for 30 min at room temperature. After washing three times with PBS, the sections were mounted with prolong gold antifade reagent with 4,6-diamidino-2-phenylindole (Thermo Fisher Scientific, Waltham, MA, USA, P36935). Fluorescent images were acquired using a Zeiss Observer. Zenpro software (version 1.0.5, Carl Zeiss, Thornwood, NY, USA) and ImageJ (1.54 g)were used to process and analyze the intensity of the Iba1/GFAP signals and lipid droplets. For the fluorescence intensity quantification of Iba1 or GFAP, two slices per mouse were imaged and the intensity was quantified for three mice in each group. Images were obtained under identical exposure conditions (40× magnitude). All Iba1+ or GFAP+ cells and LDs were detected based on the threshold fluorescence intensity of each cell, the soma diameter, and a manual counting tool within the counting frame of the regions of interest. Then, using automated measurements, the mean intensity of each soma was measured, and the data for the cocaine and saline groups were compared and are shown as fold changes. The co-localization of LDs with Iba-1 or GFAP was calculated manually on around 100 cells selected from at least six fields from three mice.

SREBP2-SiRNA transfection: siRNA transfection was performed based on the recommended protocol. In general, siRNA (20 pM) and the transfection reagent were dissolved in 100 µL of DMEM and mixed for 10 min at room temperature. The cells (80% confluent) were pre-washed with serum-free DMEM twice. The siRNA–transfection reagent mixture (in 1 mL of DMEM) was added to 6-well plates and incubated for 4 h; afterwards, another 1 mL of complete DMEM was added and the plates were incubated overnight (37 °C, 5% CO_2_). The next day, the cells were exposed to cocaine (10 µM) for another 24 h and then collected for SREBP2 WBs.

Cytotoxicity assay: Cell toxicity experiments were performed using the CyQUANT™ LDH Cytotoxicity kit (C20300, Thermo Fisher Scientific Company) following the manufacturer’s recommended protocol. In general, BV2 cells were seeded into 96-well plates (20 K/well) and incubated overnight. The next day, the cells were exposed to cocaine at different doses (1–100 µM) for 24 h.

Statistical analysis: All data are expressed as the mean ± standard error of the mean (SEM). The data were statistically evaluated using two-tailed Student’s *t*-test and one-way analysis of variance (ANOVA), followed by, when appropriate, Tukey–Kramer multiple comparisons tests using GraphPad Prism 10 (La Jolla, CA, USA). Differences with probability levels < 0.05 were considered statistically significant.

## 3. Results

### 3.1. Chronic Cocaine Administration Altered Brain Lipid Profiles

**Figure 1:**
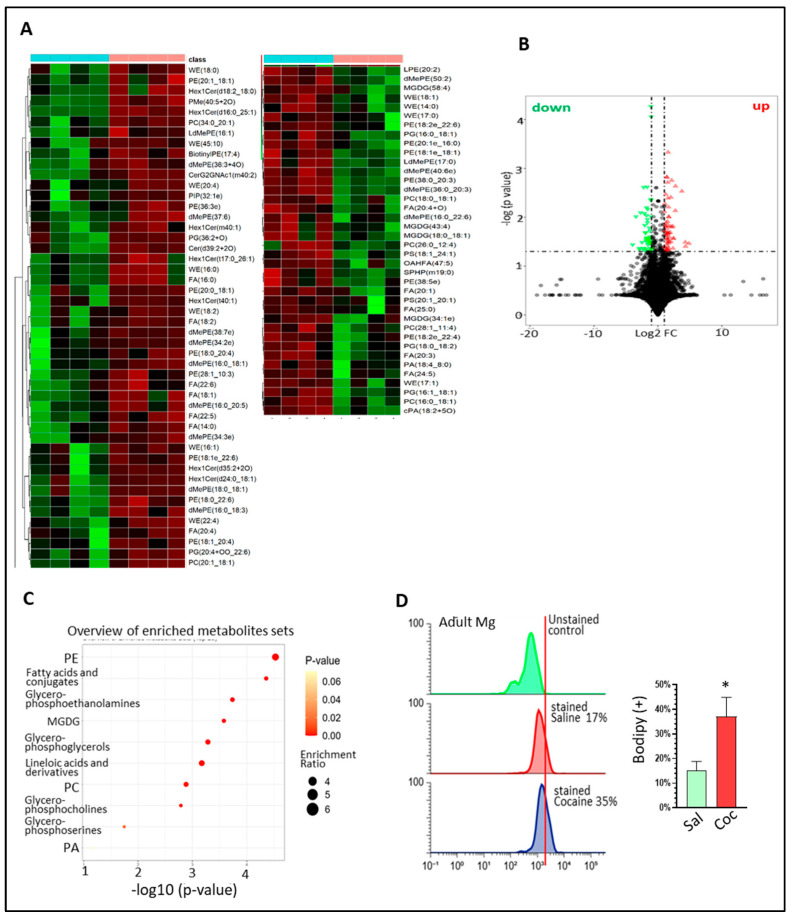
Chronic cocaine administration altered brain lipid profiles. (**A**) Hierarchical cluster analysis of saline- and cocaine-treated groups revealed up- (red) and down- (green) regulated lipids in cocaine-treated HPs (*n* = 4); (**B**) volcano plot showing up- and down- regulated lipids in cocaine-treated HPs (cutoff: 1.3 fold, *p* < 0.05); (**C**) IPA results showing enriched metabolites in cocaine-treated HPs; (**D**) flow cytometry analysis results showing percentages of BODIPY (+) adult Mg in saline- and cocaine- treated brains. The results from three independent experiments were used in the data analysis (* *p* < 0.05, two-tailed Student’s *t*-test).

To investigate the chronic effects of cocaine on brain lipid profiles, we dissected the brain hippocampi from saline- and cocaine-treated male mice (*n* = 4, 15 mg/kg i.p. daily for three weeks) for untargeted liquid chromatography–mass spectrometry (LC-MS) analysis. The lipid profiles revealed that large amounts of certain lipid species were significantly up- or down-regulated in the cocaine-treated HPs (Figure 1A: hierarchical cluster analysis; Figure 1B: volcano plot). KEGG analysis showed the most enriched metabolites, which included phosphatidylethanolamine (PE), fatty acids (FAs), glycerol–phosphoethanolamine, and monogalactosyldiacylglycerol (MGDG) (Figure 1C). These results indicate that cocaine can dysregulate lipid synthesis in vivo. Next, we assessed LD formation by assessing PLIN2 levels as well as lipid synthesis activity by monitoring the levels of SREBP1/2 and HMGCR in the brains. The results revealed that cocaine significantly increased PLIN2 levels in the HP (Supplementary Figure S1A, * *p* < 0.05). Lipid synthesis activity was also upregulated by cocaine, which was evidenced by the increased levels of nSREBP1/2 and HMGCR (Supplementary Figure S1B–D, * *p* < 0.05). We observed similar upregulation effects of cocaine on lipid synthesis activity in the prefrontal cortex (PFc) (Supplementary Figure S1E–H, * *p* < 0.05). To directly interrogate whether cocaine can affect lipid metabolism in microglia, we isolated adult microglia from the brains of both groups (*n* = 3) and stained them with BODIPY. The flow cytometry data showed that the BODIPY (+) microglia accounted for around 17% of the microglial cells in the saline-treated mice vs. 35% in the cocaine-treated mice (Figure 1D). In summary, these findings showed that chronic cocaine administration can dysregulate lipid metabolism in the brain as well as in microglia.

### 3.2. Cocaine Increased LDMA Formation and Levels of Senescent Marker p53 In Vivo

**Figure 2:**
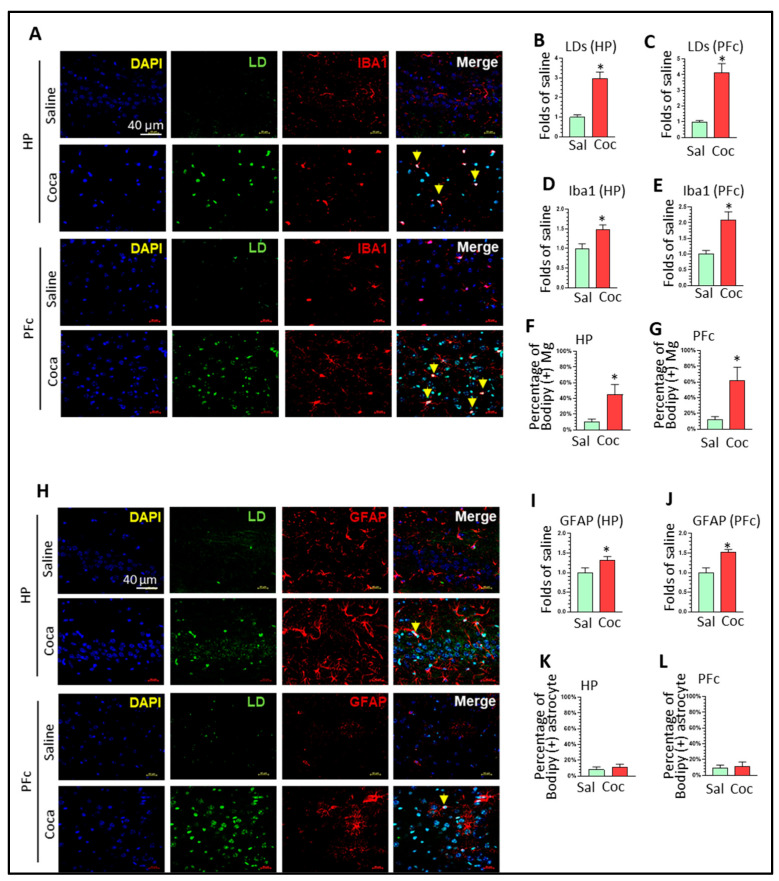
Chronic cocaine administration increased LD formation in Mg. (**A**) Immunostaining results showing the effects of cocaine on LD accumulation, Iba1 intensity, and co-localization of LDs with Iba1 in the HP and PFc of mice treated with saline or cocaine. Quantification results showing that cocaine increased (**B**,**C**) LD accumulation and (**D**,**E**) the Iba1 intensity in the HP and PFc. (**F**,**G**) Quantification results showing that cocaine increased the co-localization of LDs with Iba1 in the HP and PFc. (**H**) Immunostaining results showing the effects of cocaine on LD accumulation, GFAP intensity, and co-localization of LDs with GFAP in the HP and PFc of mice treated with saline or cocaine. (**I**,**J**) Quantification results showing that cocaine increased the GFAP intensity in the HP and PFc. (**K**,**L**) Quantification of the effects of cocaine on the co-localization of LDs with GFAP in the HP and PFc. In the immunostaining analysis, each group contained five mice, and two slices were selected from each mouse (*n* = 10). * *p* < 0.05, unpaired two-tailed *t*-test; scale bar: 40 µm. The yellow arrows indicate co-localization of the markers with LDs within Mg.

To explore the effects of cocaine on LDAM formation in vivo, we performed double immunostaining (BODIPY and Iba1) of saline- or cocaine-treated mouse brains. The results showed that cocaine significantly increased the intensity of BODIPY signaling in the HP (2.98 ± 0.32-fold) and PFc (4.12 ± 0.59-fold) (Figure 2A–C, * *p* < 0.05). Cocaine also significantly increased the intensity of Iba1 levels in the HP (1.49 ± 0.11-fold) and PFc (2.08 ± 0.27-fold), implying microglia activation, which is consistent with previous findings (Figure 2A,D,E, * *p* < 0.05) [37,38]. The percentage of LDs that co-localized with Iba1 was around 40% and 50% in cocaine-treated mice’s HP and PFc, respectively, demonstrating that cocaine can significantly increase LDMA formation (Figure 2A, yellow arrow, Figure 2F,G, * *p* < 0.05). Astrocytes are another type of glial cell and can be activated by cocaine exposure. To determine if cocaine affects LD formation in astrocytes, we performed double immunostaining of LDs and GFAP on brain cryosections. The intensity of the GFAP staining signal was upregulated by cocaine in both the HP and PFc (Figure 2H, * *p* < 0.05). However, there was no significant overlapping of LDs with GFAP (Figure 2K,L). We performed double immunostaining of LDs and NeuN (neuronal marker), and the results showed increased LD formation in the neurons of the cocaine-treated brains compared to the saline controls (Supplementary Figure S2). Overall, our findings demonstrated that cocaine can affect brain lipid metabolism and increase LD formation in both microglia and neurons.

**Figure 3:**
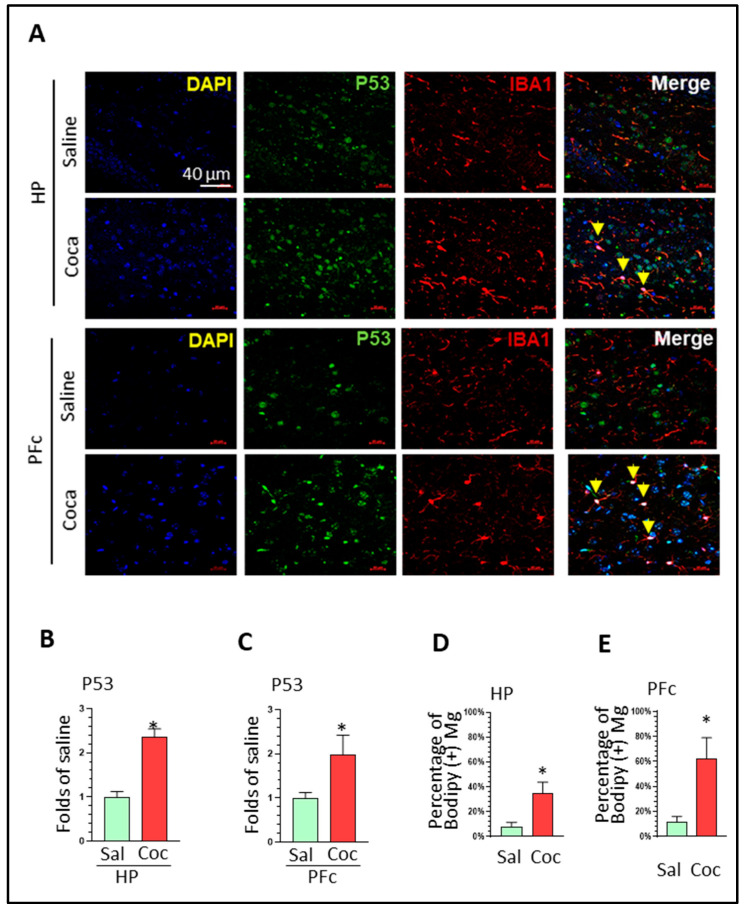
Chronic cocaine administration increased p53 levels in the brain. (**A**) Immunostaining results showing the effects of cocaine on p53 levels, Iba1 intensity, and co-localization of p53 with Iba1 in the HP and PFc of mice treated with saline or cocaine. Quantification results showing that cocaine induced p53 upregulation in the (**B**) HP and (**C**) PFc. Quantification results showing that cocaine increased the co-localization of p53 and Iba1 in the (**D**) HP and (**E**) PFc. In the immunostaining analysis, each group contained two mice, and three slices were selected from each mouse (*n* = 6). * *p* < 0.05, unpaired two-tailed *t*-test; scale bar: 40 µm. The yellow arrows indicate the co-localization of the markers with LDs within Mg.

LDAMs share common features with senescent cells. To explore whether cocaine can also increase senescent markers in microglia, we performed double immunostaining of p53 and Iba1 on the brain sections from the two groups. The results showed that cocaine significantly increased p53 levels in both the HP (2.53 ± 0.19-fold, * *p* < 0.05) and PFc (1.98 ± 0.44-fold, * *p* < 0.05) (Figure 3A–C). In addition, the co-localization of p53 and Iba1 significantly increased in cocaine-treated brains (Figure 3D, * *p* < 0.05) and PFcs (Figure 3E, * *p* < 0.05). These results implied that LDAMs are associated with senescent microglia when exposed to cocaine in vivo. However, p53 is not a specific marker of cellular senescence. P53 can be activated in multiple non-senescent stress responses, including DNA damage and oxidative stress [39]. Thus, a decisive conclusion on the association between LDAMs and senescent microglia requires further investigation.

### 3.3. Cocaine Increased LD Formation and Induced Senescence in Microglia In Vitro

**Figure 4:**
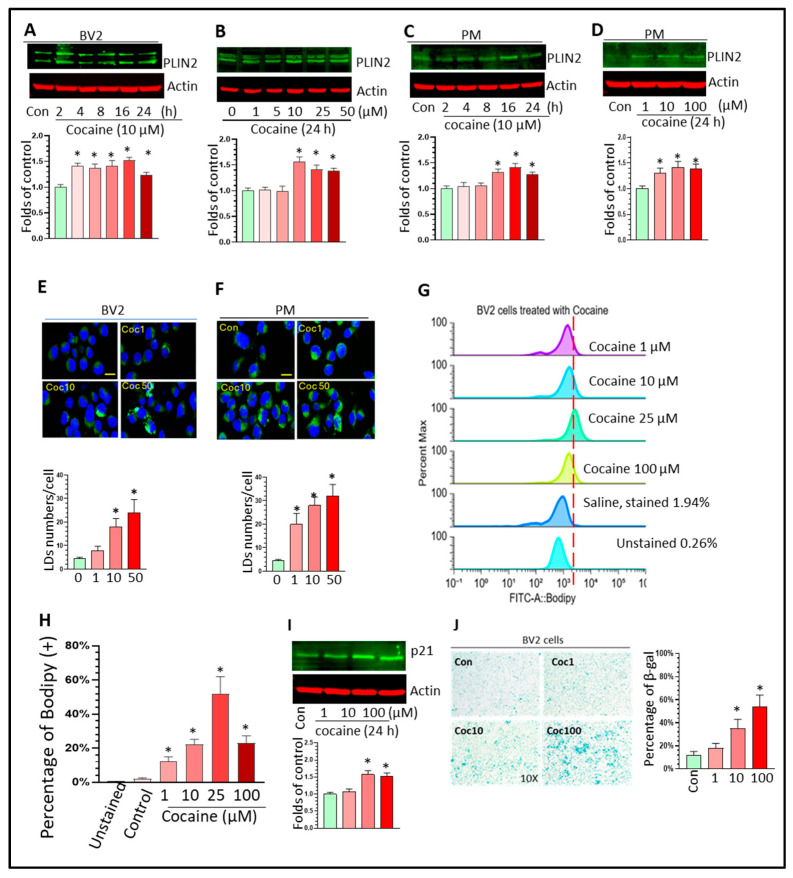
Cocaine upregulated PLIN2 levels, increased LD formation, and induced senescence in Mg in vitro. Cocaine increased PLIN2 levels in (**A**,**B**) BV2 cells and (**C**,**D**) PMs in both time-course and dose–response experiments. (**E**,**F**) Cocaine increased LD formation in both BV2 and PMs. (**G**,**H**) Flow cytometry analysis showing that cocaine increased the percentage of BODIPY (+) Mg in vitro. Cocaine increased the levels of (**I**) the senescent marker p21 and (**J**) β-gal staining in BV2 cells in a dose-dependent manner. In the immunostaining analysis, around 100 cells for each condition were analyzed. For the WBs, flow cytometry experiments, and β-gal staining, the results from three independent experiments were used in the data analysis. * *p* < 0.05, one-way ANOVA for multiple-group comparisons. The original Western blot images can be found in Supplementary Figure S6.

To explore whether cocaine can also increase LD accumulation in vitro, we monitored the PLIN2 levels in BV2 cells treated with different cocaine concentrations. First, we plotted the dose–response curve to determine the most suitable doses of cocaine for the in vitro studies. BV2 cells were seeded into 96-well plates and exposed to different doses (1–100 µM) for 24 h. The cytotoxicity results showed that cocaine up to 10 µM did not induce significant cell death (* *p* < 0.05, Supplementary Figure S3). We then treated BV2 cells with cocaine in time-course and dose–response experiments. The results showed that cocaine increased PLIN2 levels at 16 h post-treatment and the upregulation effects persisted up to 24 h post-treatment (Figure 4A, * *p* < 0.05). Cocaine also significantly enhanced PLIN2 levels at multiple doses (10–50 µM, Figure 4B, * *p* < 0.05). We obtained similar upregulation trends for PLIN2 levels in PMs with various cocaine concentrations (Figure 4C,D, * *p* < 0.05). BODIPY staining validated the cocaine-induced increase in LD formation in both types of cells (Figure 4E,F, * *p* < 0.05). In addition, flow cytometry analyses revealed that cocaine can dose-dependently increase the percentage of BODIPY (+) BV2 cells (Figure 4G,H, * *p* < 0.05). The BODIPY (+) BV2 cell ratio also decreased at a dose of 100 µM. It is possible that BV2 cells die when exposed to high cocaine doses and the flow cytometry analysis only counted living and healthy BV2 cells.

Next, we verified whether LDAMs are also associated with senescent status in vitro. We monitored the levels of p21, which is a downstream effector of p53 and induces cell cycle arrest [40]. The results showed that cocaine dose-dependently increased p21 expression (Figure 4I, * *p* < 0.05) and the intensity of the β-gal signal in BV2 cells (Figure 4J, * *p* < 0.05). These results suggest that LDAMs are closely associated with the senescent status of microglia under cocaine exposure.

### 3.4. Cocaine Upregulated SREBP-Mediated Lipid Synthesis Activity in Microglia In Vitro

**Figure 5:**
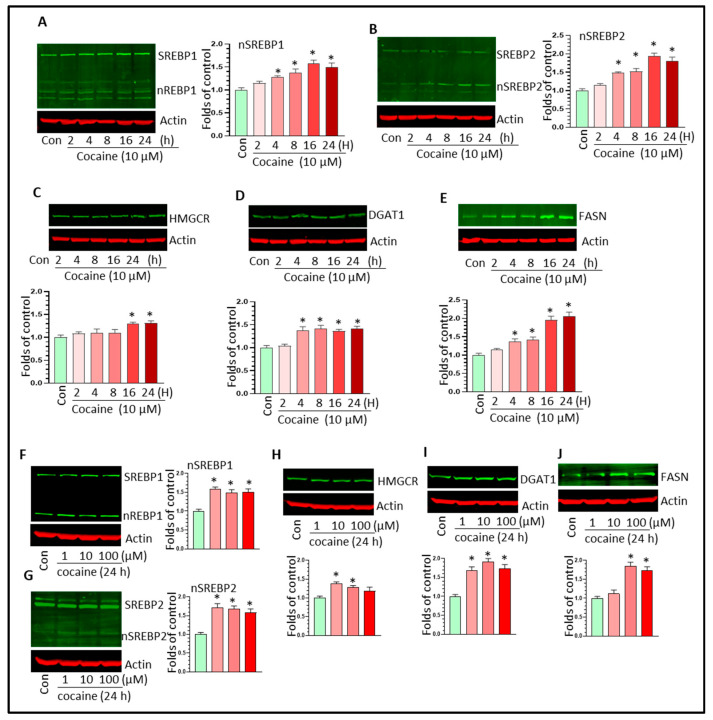
Cocaine upregulated SREBP-mediated lipid synthesis activity in BV2 cells. Cocaine increased (**A**) nSREBP1; (**B**) nSREBP2; and (**C**–**E**) HMGCR, DGAT1, and FASN levels in BV2 cells over time. Cocaine dose-dependently increased (**F**) nSREBP1, (**G**) nSREBP2, (**H**–**J**) HMGCR, DGAT1, and FASN levels in BV2 cells. For the WB analysis, three independent experiments were performed. * *p* < 0.05, one-way ANOVA for multiple-group comparison. The original Western blot images can be found in Supplementary Figure S8.

**Figure 6:**
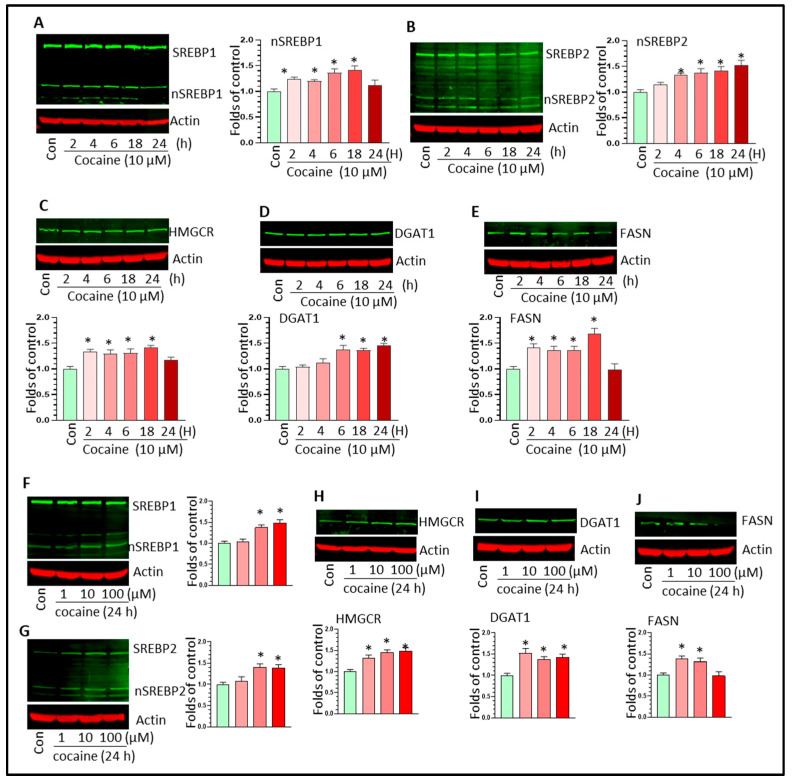
Cocaine upregulated SREBP-mediated lipid synthesis activity in PMs. Cocaine increased (**A**) nSREBP1, (**B**) nSREBP2, (**C**–**E**) HMGCR, DGAT1, and FASN levels in PMs over time. Cocaine dose-dependently increased (**F**) nSREBP1, (**G**) nSREBP2, (**H**–**J**) HMGCR, DGAT1, and FASN levels in PMs. For the WB analysis, three independent experiments were performed. * *p* < 0.05, one-way ANOVA for multiple-group comparison. The original Western blot images can be found in Supplementary Figure S9.

To explore the mechanisms underlying cocaine-mediated LD formation in vitro, we assessed the effects of cocaine on SERBP-mediated lipid synthesis pathway activity in microglia. In addition to monitoring the levels of SREBP1/2 and HMGCR, we also assessed the levels of fatty acid synthase (FASN) and diacylglycerol O-acyltransferase 1 (DGAT1) in cocaine-treated BV2 cells. In the time-course experiments, cocaine upregulated the levels of nSREBP1 at 4 h after treatment and this upregulation was sustained until 24 h post-treatment (Figure 5A, * *p* < 0.05). We observed similar upregulation effects of cocaine on nSREBP2 (Figure 5B, * *p* < 0.05). Cocaine also increased the levels of HMGCR, DGAT1, and FASN in BV2 cells in a time-dependent manner (Figure 5C–E; * *p* < 0.05). Cocaine at different doses also enhanced nSREBP1/2 levels in all groups (Figure 5F,G; * *p* < 0.05). Similarly, the levels of HMGCR, DGAT1, and FASN were also increased by cocaine in a dose-dependent manner (Figure 5H–J; * *p* < 0.05). For further validation, we investigated lipid synthesis activity in cocaine-treated PMs. Cocaine upregulated the levels of nSREBP1/2 in a time-dependent manner (Figure 6A,B; * *p* < 0.05). The levels of HMGCR, DGAT1, and FASN also increased in cocaine-exposed PMs over time (Figure 6C–E; * *p* < 0.05). In PMs, different doses of cocaine also increased nSREBP1/2 (Figure 6F,G, * *p* < 0.05) and HMGCR, DGAT1, and FASN levels (Figure 6H–J; * *p* < 0.05). To validate the role of SREBP in cocaine-mediated LD upregulation, we assessed Plin2 levels in BV2 cells with SREBP2 knockdown. Our results showed that SREBP2 knockdown significantly mitigated the cocaine-mediated upregulation of Plin2 levels (* *p* < 0.05, Supplementary Figure S4). These results confirmed that SREBP2 plays a critical role in cocaine-mediated LD formation.

### 3.5. Cocaine Upregulated SREBP-Mediated Lipid Synthesis Activity in HMC3 Cells

**Figure 7:**
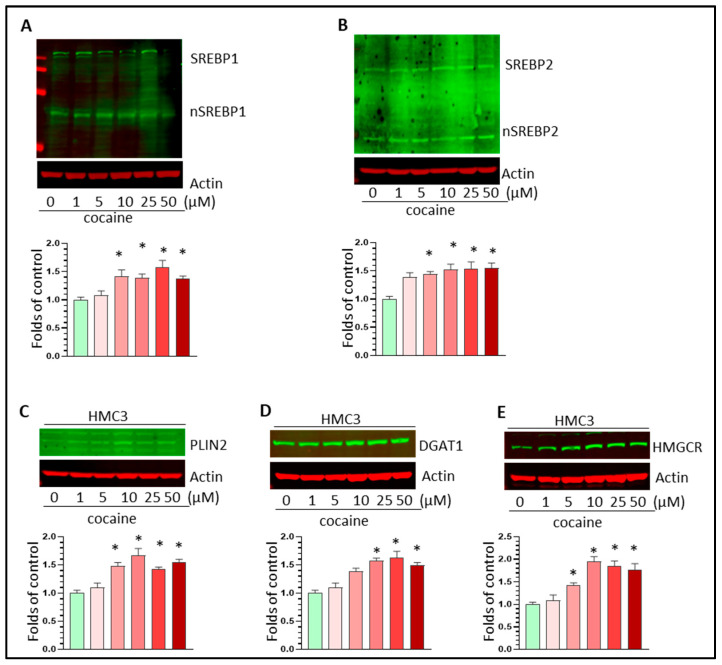
Cocaine upregulated SREBP-mediated lipid synthesis activity in HMC3 cells. Cocaine dose-dependently increased (**A**) nSREBP1, (**B**) nSREBP2, and (**C**–**E**) Plin2, DGAT1, and HMGCR levels in HMC3 cells. For the WB analysis, three independent experiments were performed. * *p* < 0.05, one-way ANOVA for multiple-group comparison. The original Western blot images can be found in Supplementary Figure S10.

To explore whether cocaine could also affect SREBP-mediated lipid synthesis in human-derived microglia, we treated HMC3 cells with varying doses of cocaine (1–50 µM) for 24 h, which were then subjected to WB analysis for SREBP1/2, Plin2, DGAT1, and HMGCR. The results showed that cocaine can increase the levels of these targeted molecules in a dose-dependent manner (* *p* < 0.05, Figure 7A–E). Our results imply that cocaine can increase LD formation in human-derived microglia.

## 4. Discussion

In this study, we explored the effects of cocaine on lipid metabolism in both rodent- and human-derived microglia and demonstrated that cocaine could increase lipid synthesis activity, leading to increased LDAM formation in vitro and in vivo. Mechanistically, cocaine upregulated the activity of the SREBP-mediated lipid synthesis pathway. We also found that increased LD formation was associated with microglial senescence when they are exposed to cocaine. Since LDAMs are characterized by sustained inflammation and are associated with senescent microglia, these results imply a novel immunometabolic mechanism underlying the age-related neurological syndromes in people with addictions. LDAM removal might serve as a promising therapeutic approach to mitigate the pathogenesis of cocaine use disorders (CUDs).

One of our interesting findings was that cocaine increased LD formation in Mg and neurons in vivo. We performed double immunostaining of Iba1/BODIPY, GFAP/BODIPY, and NeuN/BODIPY in the brains of mice subjected to chronic cocaine administration. The LDs mainly colocalized with Iba1 and neurons but not with GFAP. Previous studies have shown that LDs can be found in astrocytes in various diseases models [41,42]. The mechanism underlying cocaine’s cell-type-specific effects on LD accumulation in vivo remains mostly unknown. Cocaine can induce astrocyte activation [43]; therefore, immune responses cannot explain the differences in LD accumulation between microglia and astrocytes. Possible explanations could be due to the intrinsic properties of microglia and astrocytes and the simulation intensity. Microglia are brain immunocompetent macrophages that are ready to elicit immune responses in response to various internal and external stimuli. Therefore, microglia are very sensitive to subtle environmental changes. Astrocytes are mainly supporting cells and provide energy and nutrients to maintain normal neuronal functions. Three weeks of cocaine injections, compared with other stimuli such as stroke or brain injuries, might be too mild and not strong enough to change lipid metabolism in astrocytes and we cannot exclude the possibility that we would observe LD accumulation in astrocytes with long-term or with higher doses of cocaine injection. We also observed significant increases in LDs in neurons after cocaine administration. The biological effects of this phenomenon remain unknown and deserve further investigation. Here we showed increases in LDAMs in cocaine-treated HPs and PFcs. We also monitored LDAM formation in the striatum, which is another region sensitive to cocaine exposure. However, we did not observe significantly increased LDAM counts in this region. The striatum is critical for cocaine-mediated behavioral changes at earlier phases (acute response) and the HP and PFc are more relevant to the cognitive and memory deficiencies induced by cocaine [44]. This region-specific distribution of LDAMs implied that lipid metabolism dysregulation may be more relevant to cocaine-mediated memory and cognitive decline, which is consistent with previous findings that LDAMs are closely associated with aging-related neurological syndrome in neurodegenerative diseases.

CUDs are brain diseases characterized by abnormal microglia activation. Previous investigations have shown that cocaine can alter the brain lipid profile, implicating lipid metabolism in the development of CUDs [25,26]. Here, we demonstrated that cocaine can dysregulate lipid metabolism and increase PLIN2 levels in microglia, indicating that increased LDAM formation might be responsible for the pathogenesis of CUDs. Our findings may have a broader significance. Since various types of abused drugs, including Meth, morphine, and alcohol, have been shown to induce brain lipid metabolism dysregulation [31,32,35,36], we extrapolate that LDAM accumulation could be a common event in substance use disorders (SUDs) and immunometabolism in involved in SUD pathogenesis; however, this hypothesis requires further investigation. Cocaine increased LDAM formation and led to senescent microglia. These results are consistent with previous findings that LDAMs share common features with senescent cells including phagocytic defects, excessive secretion of proinflammatory cytokines, high levels of reactive oxygen species, and reductions in cholesterol efflux [11,13,45]. Therefore, LDAMs might be senescent microglia induced by abused drugs, which then trigger the accelerated brain aging observed in SUDs.

In searching for mechanisms responsible for cocaine-mediated LD formation, we focused on the activity of the SERBP-mediated lipid synthesis pathway. We demonstrated that cocaine increased the levels of nSREBP1/2, DGAT1, and FASN in microglia in vitro and in vivo, implying that increased synthesis activity plays a critical role in LD formation. However, we understand that this proposed mechanism needs further validation through pharmacological (inhibitor) or genetic approaches (siRNA knockdown). LDs are dynamic intracellular organelles and their formation is regulated by multiple processes including lipid synthesis, influx and efflux, and degradation [21]. The degradation process includes lipophagy (a specific form of autophagy) and lipolysis, which is carried out by lipases [45,46]. Adipose triglyceride lipase (ATGL), hormone-sensitive lipase (HSL), and monoglyceride lipase (MGL) are three enzymes that break down TAGs into FAs [47]. We monitored the levels of these three lipases in BV2 cells and PMs under the cocaine treatments and did not find significant changes in their levels, indicating that lipase activity is not significantly affected by cocaine. Our previous investigations have shown that cocaine can interrupt autophagy processes, leading to Mg activation [37,38]. Therefore, it is possible that lipophagy disruption is involved in cocaine-mediated LD formation in microglia.

Our investigation has some limitations. We employed a three-week injection regimen to investigate the chronic effects of cocaine on brain lipid metabolism and LDAM formation. The administration approach (passive injection vs. self-administration) may have different effects on brain cells and immune responses [48]. The best approach to investigate the biological effects of cocaine in the brain is through self-administration, where the rodents take cocaine voluntarily, mimicking human behaviors [49,50]. To consolidate the findings showing that LDAMs are involved in the pathogenesis of CUDs, we will employ a self-administration protocol in mice to explore the effects of cocaine on lipid metabolism processes and LDAM formation in future studies.

## 5. Conclusions

In conclusion, we demonstrated that cocaine can dysregulate lipid metabolism and increase LDAMs through increasing SREBP-mediated lipid synthesis activity. Our findings provide evidence that LDAMs could be involved in the pathogenesis of CUDs. Targeting lipid metabolism and LDAM removal could be a novel therapeutic approach for alleviating aging-related neurological syndromes in people with cocaine addictions.

## Data Availability

The original contributions presented in this study are included in the article/Supplementary Materials. Further inquiries can be directed to the corresponding author.

## Supplementary Materials

The following supporting information can be downloaded at: https://www.mdpi.com/article/10.3390/biom16040526/s1; Figure S1: Chronic cocaine administration increased the activity of SREBPs-mediated synthesis pathway; Figure S2: The colocalization of LDs with neurons. NeuN and Iba1 double immunostaining was performed in the brain sections of saline- and cocaine- treated mice. Each group 2 contained five mice, and two slices were selected from each mouse (*n* = 10). (Scale bar 40 μm). The yellow arrows indicated the co-localization of LDs within neuron; Figure S3: The cytotoxicity of cocaine BV2 cells. BV2 cells were seeded into 96-well plates and exposed to cocaine at varying doses (1–100 μM). The cytotoxicity was determined by CyQUANT™ LDH Cytotoxicity kit. The results showed that cocaine at doses of 50 and 100 μM could induce significant cell loss (* *p* < 0.05, one-way ANOVA, experiments were repeated five times for statistical analysis); Figure S4: SREBP2 knockdown mitigated cocaine-mediated LDs formation in BV2 cells; Figure S5–S10: Original Western blot images.
