# Supplementary material for: Cocaine Upregulates Microglial Lipid Droplet Formation Through Increasing Lipid Synthesis Activity In Vitro and In Vivo
Source: Biomolecules. 2026 Apr 1;16(4):526. doi: 10.3390/biom16040526 (PMC13114159; doi:10.3390/biom16040526)
Supplement: Supplementary file 1 [file biomolecules-16-00526-s001.zip › biomolecules-4066216-supplementary.pdf]

Supplementary Materials

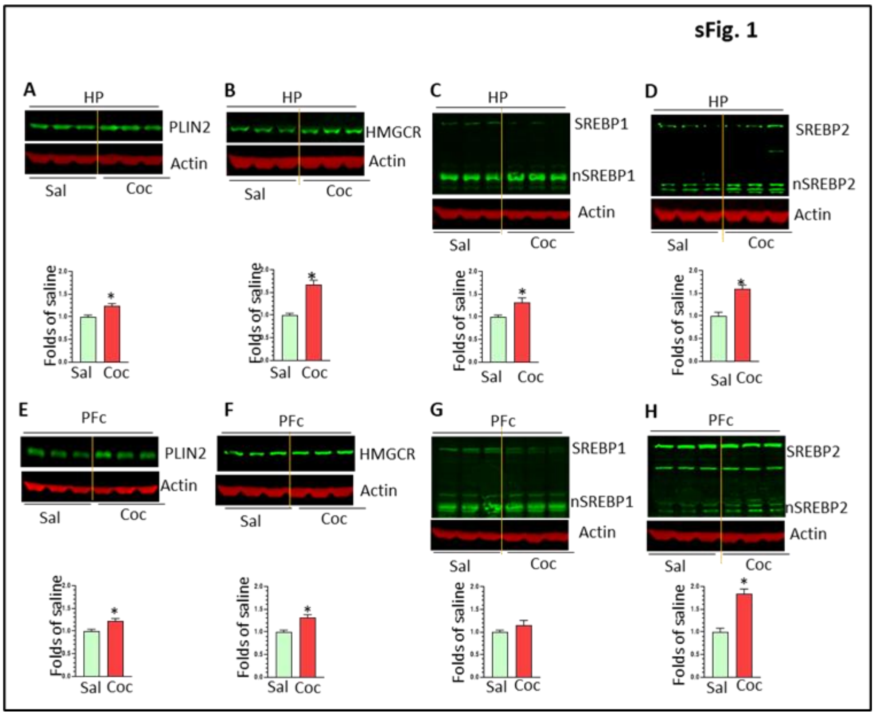

Supplementary Figure S1: Chronic cocaine administration increased the activity of SREBPs-mediated synthesis pathway. (A - D) Chronic cocaine administration increased the levels of PLIN2, HMGCR, nSREBP1, and nSREBP2 in the HP; (E - H) Chronic cocaine administration increased the levels of PLIN2, HMGCR, nSREBP1, and nSREBP2 in the HP. (n = 3, * p < 0.5, two-tails student-t tests)The original Western blot images can be found in theSupplementary Figure 5.

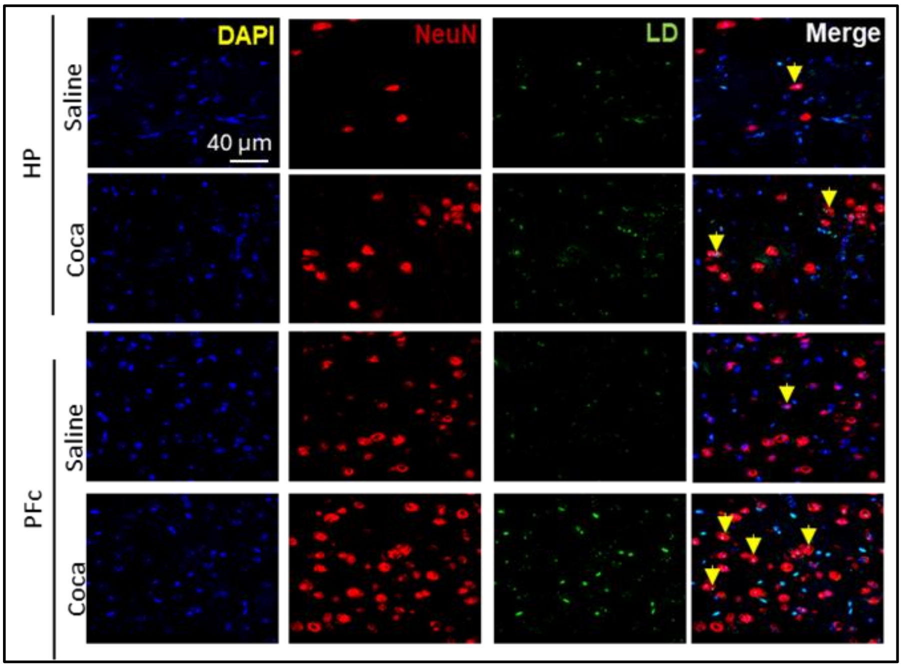

Supplementary Figure S2: The colocalization of LDs with neurons. NeuN and Iba1 double immunostaining was performed in the brain sections of saline- and cocaine- treated mice. Each group

contained five mice, and two slices were selected from each mouse (n = 10). (Scale bar 40 μm). The yellow arrows indicated the co-localization of LDs within neuron.

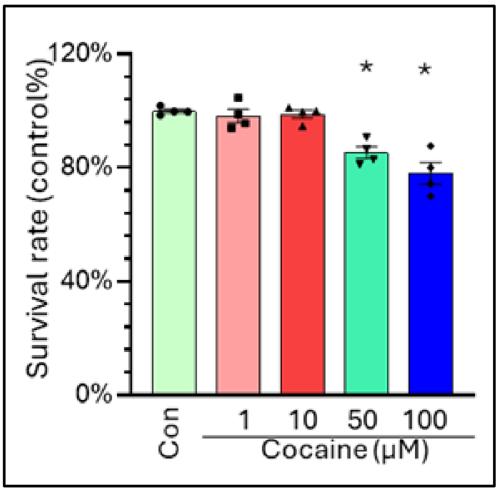

Supplementary Figure S3: The cytotoxicity of cocaine BV2 cells. BV2 cells were seeded into 96-well plates and exposed to cocaine at varying doses (1 - 100 μM). The cytotoxicity was determined by CyQUANT™ LDH Cytotoxicity kit. The results showed that cocaine at doses of 50 and 100 μM could induce significant cell loss (* $p < 0.05$, one-way ANOVA, experiments were repeated five times for statistical analysis).

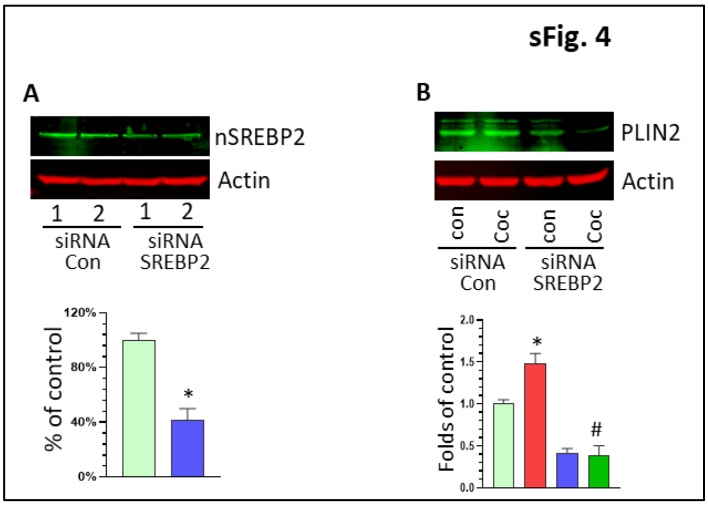

Supplementary Figure S4: SREBP2 knockdown mitigated cocaine-mediated LDs formation in BV2 cells**.** (**A**) Validation on the transfection of SREBP2-siRNA decreased nSREBP2 levels in BV2 cells (* $p < 0.05$ vs. controls, t-test); (**B**) SREBP2 knockdown mitigated cocaine-mediated Plin2 in BV2 cells (* $p < 0.05$ vs. controls; # $p < 0.05$, SREBP2-siRNA + cocaine vs. control siRNA + cocaine, one-way ANOVA. Experiments were repeated four times for statistical analysis).The original Western blot images can be found in theSupplementary Figure 7.

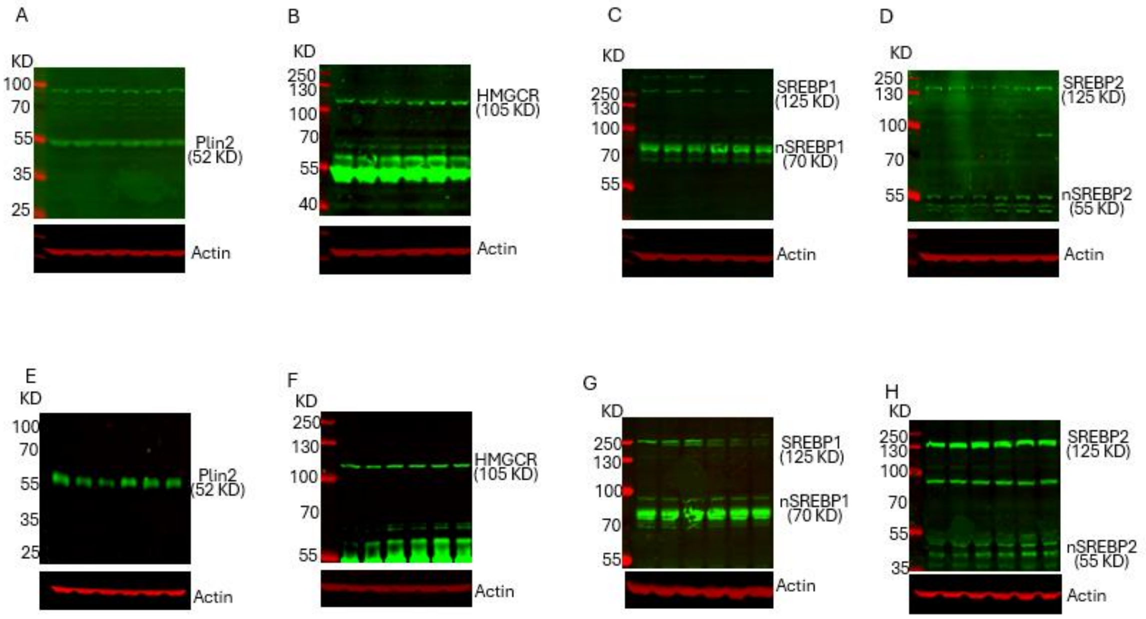

Supplementary Figure S5: Original Western blot plot of Supplementary Figure 1.

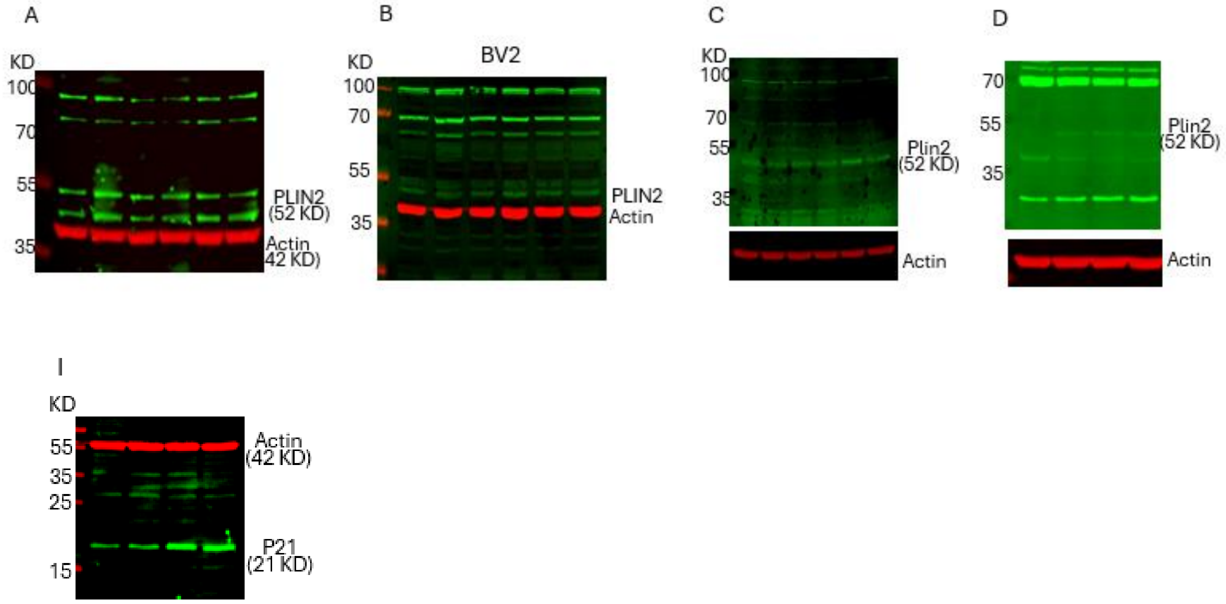

Supplementary Figure S6: Original Western blot plot of Figure 4

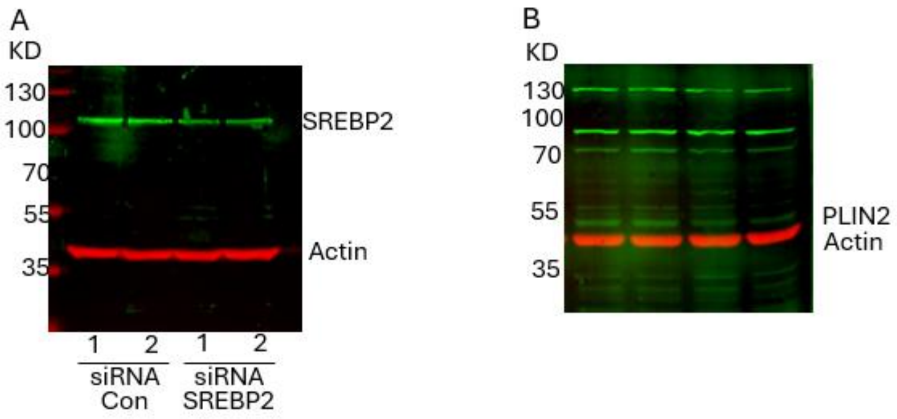

Supplementary Figure S7: Original Western blot plot of Supplementary Figure 4

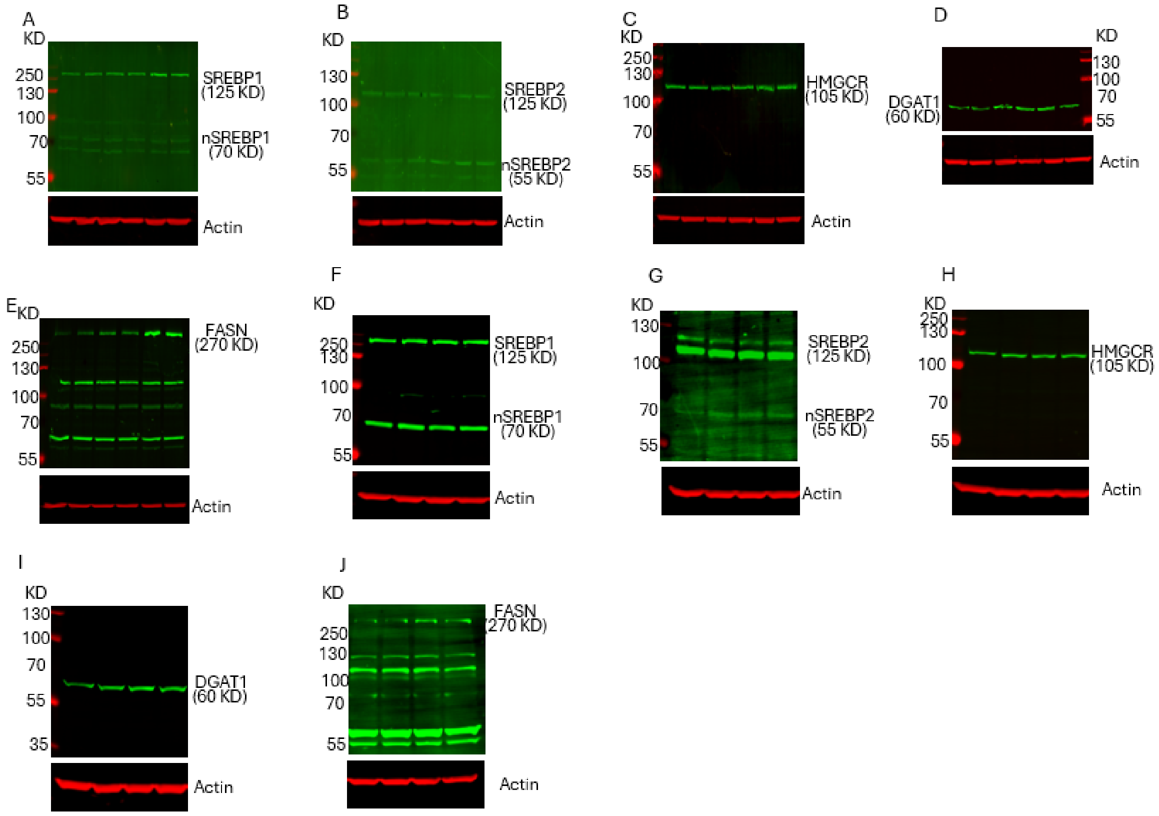

Supplementary Figure S8: Original Western blot plot of Figure 5

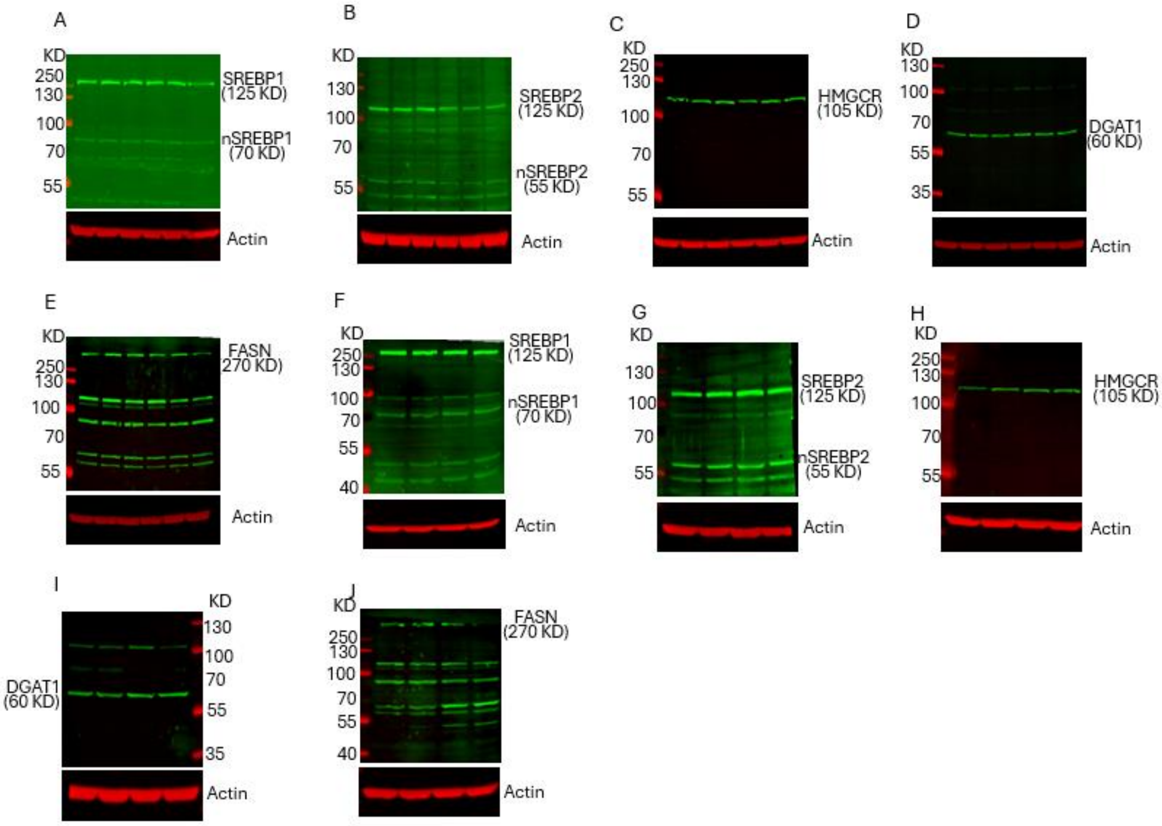

Supplementary Figure S9: Original Western blot plot of Figure 6

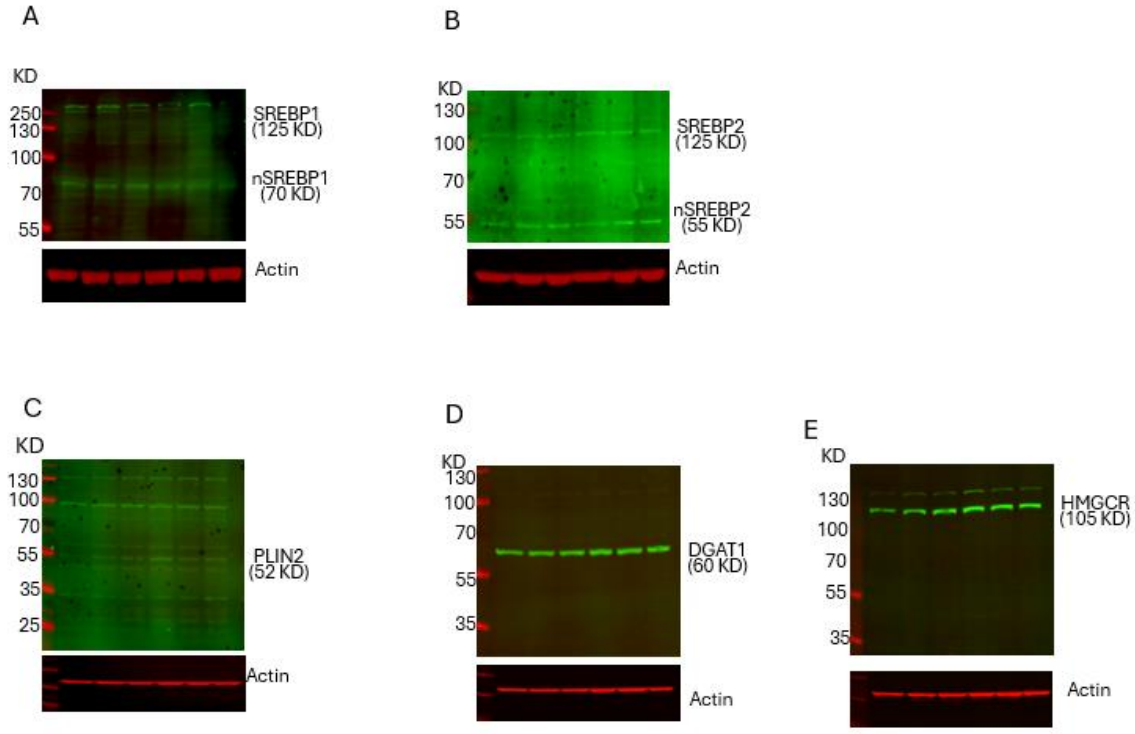

Supplementary Figure S10: Original Western blot plot of Figure 7
